# Age-Related Incidence and Peak Occurrence of Contralateral Breast Cancer

**DOI:** 10.1001/jamanetworkopen.2023.47511

**Published:** 2023-12-15

**Authors:** Hakyoung Kim, Tae In Yoon, Seonok Kim, Sae Byul Lee, Jisun Kim, Il Yong Chung, Beom Seok Ko, Jong Won Lee, Byung Ho Son, Young Jin Lee, Sungchan Gwark, Hee Jeong Kim

**Affiliations:** 1 Division of Breast Surgery, Department of Surgery, University of Ulsan College of Medicine, Asan Medical Center, Seoul, Republic of Korea; 2 Department of Surgery, Dongguk University College of Medicine, Dongguk University Ilsan Hospital, Goyang, Republic of Korea; 3 Division of Breast Surgery, Department of Surgery, Dongnam Institute of Radiological and Medical Science, Busan, Republic of Korea; 4 Department of Clinical Epidemiology and Biostatistics, College of Medicine, University of Ulsan, Asan Medical Center, Seoul, Republic of Korea; 5 Department of Surgery, Ewha Woman’s University College of Medicine, Ewha Woman’s University Mokdong Hospital, Seoul, Republic of Korea

## Abstract

**Importance:**

Young patients with breast cancer have higher risk for developing contralateral breast cancer (CBC) and have epidemiologic characteristics different from those of older patients.

**Objective:**

To examine the incidence and peak occurrence of CBC according to age at primary breast cancer (PBC) surgery.

**Design, Setting, and Participants:**

This cohort study included patients who were diagnosed with and underwent surgery for unilateral nonmetastatic breast cancer at Asan Medical Center, Korea, between January 1, 1999, and December 31, 2013, with follow-up through December 31, 2018. Data were analyzed from December 1, 2021, through April 30, 2023. Patients were divided into 2 groups according to their age at surgery for PBC: younger (≤35 years) vs older (>35 years).

**Main Outcomes and Measures:**

The main outcomes were cumulative incidence and hazard rate of CBC in the entire study population and in subgroups divided by cancer subtype, categorized according to hormone receptor (HR) and *ERBB2* status.

**Results:**

A total of 16 251 female patients with stage 0 to III breast cancer were analyzed; all patients were Korean. The mean (SD) age was 48.61 (10.06) years; 1318 patients (8.11%) were in the younger group, and 14 933 (91.89%) were in the older group. Median follow-up was 107 months (IQR, 79-145 months). Compared with the older group, the younger group had significantly higher incidence of CBC (10-year cumulative incidence, 7.1% vs 2.9%; *P* < .001) and higher risk (hazard ratio, 2.10; 95% CI, 1.62-2.74) of developing CBC. The hazard rate, which indicates risk for developing CBC at a certain time frame, differed according to the subtype of primary cancer. In patients with the HR+/*ERBB2*– subtype, the risk increased continuously in both age groups. In patients with the triple negative subtype, the risk increased until approximately 10 years and then decreased in both age groups. Meanwhile, in the HR−/*ERBB2*+ subtype, risk peaked earlier, especially in the younger group (1.7 years since first surgery in the younger group and 4.8 years in the older group).

**Conclusions and Relevance:**

In this cohort study, patients aged 35 years or younger with breast cancer had a higher risk of developing CBC than older patients. Moreover, young patients with the HR−/*ERBB2*+ subtype tended to have a shorter interval for developing CBC. These findings might be useful in guiding treatment decisions, such as contralateral prophylactic mastectomy.

## Key Points

**Question:**

Do the cumulative incidence and peak occurrence period of contralateral breast cancer (CBC) differ according to age at primary breast cancer surgery?

**Findings:**

In this cohort study of 16 251 patients with stage 0 to III breast cancer, the younger group (≤35 years) had significantly higher incidence of CBC than the older group (>35 years). The peak occurrence period for CBC was earlier in the younger group vs the older group with hormone receptor–negative, *ERBB2*-positive subtype.

**Meaning:**

The findings provide information that may be valuable when evaluating risk for CBC.

## Teaser

This cohort study compares the cumulative incidence and peak occurrence period of contralateral breast cancer between patients aged 35 years or younger and those older than 35 years at the time of surgery for primary breast cancer.

## Introduction

Improved survival for patients with breast cancer coupled with advances in treatment have resulted in higher numbers of patients developing contralateral breast cancer (CBC).^1^ The cumulative incidence rate for development of CBC at 5 to 10 years and more than 10 years of follow-up ranges from 1.15% to 3.8% and 3.8% to 8.3%, respectively.^2^ Factors associated with developing CBC include diagnosis of primary breast cancer (PBC) at a younger age, family history of breast cancer, breast cancer gene (*BRCA*) sequence variations, overweight, large tumor size, negative hormone receptor (HR) status, lobular type, and not undergoing adjuvant endocrine therapy or chemotherapy.^3,4^ Diagnosis of PBC at a younger age is a significant risk factor. Kurian et al^5^ reported that women diagnosed with PBC before age 30 years had a 36-fold higher risk of developing CBC than those at any other age. Similarly, a variety of studies have shown younger age groups to have elevated risk of CBC (2- to 36-fold).^6,7,8^ Suggested reasons for the high incidence of CBC development in younger patients with breast cancer are genetic factors such as family history or *BRCA* variation, a high prevalence of triple-negative breast cancer, and the aggressive character of PBC.^7,9,10^

Meanwhile, the rate of contralateral prophylactic mastectomy (CPM) is increasing, especially in young patients.^11^ As a higher proportion of younger patients was observed in the age distribution of Korean patients with breast cancer compared with those in Western countries,^12,13^ it is important to identify and educate patients who develop breast cancer at a young age about the risk of developing CBC before deciding on a treatment option. A previous study by some of us^9^ investigating age-related risk factors for developing CBC using a propensity-matched cohort (age <35 vs ≥35 years) concluded that patients younger than 35 years had 2.5 times the risk of CBC development compared with patients aged 35 years or older. In the younger cohort, patients with *ERBB2*+ subtype PBC and family history of breast cancer had increased risk of developing CBC. Additionally, in the younger cohort, the hazard rate (incidence in a certain time frame) of CBC differed according to the PBC subtype. Patients with *ERBB2* overexpression tended to develop CBC in a shorter time interval compared with patients who had other subtypes; patients with the *ERBB2*+ subtype had the highest incidence of developing CBC at 4.6 years after surgery for PBC, and the incidence among patients with the HR+/*ERBB2*– subtype peaked at 7.1 years.^9^

In this study, we aimed to evaluate the risk of developing CBC in younger patients by comparing incidence of CBC segregated by age at surgery for PBC. In addition, we examined the varying interval for developing CBC in younger patients (age ≤35 years) vs older patients (age >35 years).

## Methods

### Study Population

In this retrospective, single-center cohort study, we used the Asan database, a prospectively collected archive of patients with breast cancer treated at the Asan Medical Center, Korea, that provides information on patient age, clinical manifestations, pathology reports, types and modality of treatment, types of recurrence, and follow-up period. This study was approved by the Asan Medical Center review board. Informed consent was waived because the study was based on retrospective clinical data. We followed the Strengthening the Reporting of Observational Studies in Epidemiology (STROBE) reporting guideline.

**Figure 1.**
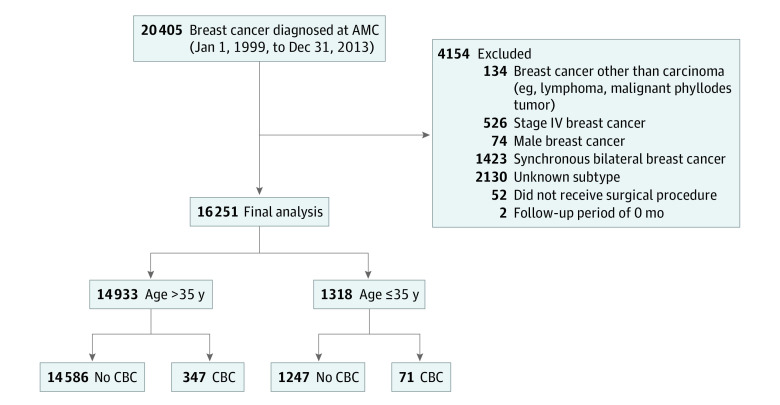
Study Population AMC indicates Asan Medical Center; CBC, contralateral breast cancer.

We collected data on patients who were diagnosed with breast cancer at Asan Medical Center between January 1, 1999, and December 31, 2013. Patients with other malignant breast diseases, such as phyllodes tumor or lymphoma; patients with stage IV breast cancer; male patients with breast cancer; and patients with synchronous bilateral breast cancer were excluded. Synchronous bilateral breast cancer was defined as the simultaneous detection of CBC within 6 months after the diagnosis of PBC. From these data, we excluded patients with unknown subtype, patients who did not receive surgery, and patients with no follow-up (Figure 1). We obtained clinicopathological data for PBC, including patient age at surgery, body mass index (BMI; calculated as weight in kilograms divided by height in meters squared), family history, *BRCA* variation status, pathology, and treatment modality.

### Assessment

We analyzed whether patients who met the criteria developed CBC during follow-up. Next, we analyzed factors associated with developing CBC in the whole study population and in the subset of patients who underwent a BRCA test. Diagnosis of CBC was defined as the first occurrence of cancer more than 6 months after surgery for PBC. In all patients, follow-up was done from the time of PBC surgery to the earliest of the following: time of CBC surgery; any kind of recurrence; death; last outpatient department visit before December 31, 2018; or maximum follow-up (defined as 17 years).

To compare the incidence and hazard rate of CBC development by age group, we divided patients into 2 groups based on their age at surgery for PBC: younger group (age ≤35 years) vs older group (age >35 years). In these 2 groups, we compared cumulative incidence in the whole population and by subgroup according to PBC subtype. We also analyzed hazard rates (ie, the risk for developing CBC within a certain time frame) according to age group in the whole population and by subgroup according to PBC subtype.

### Pathology

Pathologic data were evaluated at the Department of Pathology at the Asan Medical Center. We used pathologic TNM staging for patients who had up-front surgery and clinical TNM staging for those who underwent neoadjuvant chemotherapy. TNM stage was assigned according to the *American Joint Committee on Cancer Staging Manual* (7th edition) classification. Tumor subtypes were categorized according to HR and *ERBB2* status as HR+/*ERBB2*−, HR+/*ERBB2*+, HR−/*ERBB2*+, and HR−/*ERBB2*−. Hormone receptor was considered positive if either estrogen or progesterone receptor was positive. Immunohistochemistry was used to determine the estrogen HR and *ERBB2* status. Estrogen and progesterone receptor statuses were considered positive if more than 10% of the cells were positive. For *ERBB2* overexpression, patients with immunohistochemistry grades 0 or 1 were considered negative and those with grade 3 or higher were considered positive. Patients with grade 2 were further evaluated using fluorescence in situ hybridization.

### Statistical Analysis

All statistical analyses were performed using SAS, version 9.4 (SAS Institute Inc) and R, version 3.6.1 (R Project for Statistical Computing). To compare clinicopathological variables between the younger and older groups, the χ^2^ test was used for categorical variables and the Mann-Whitney *U* test for continuous variables. A Cox proportional hazards regression model was used to identify factors associated with CBC. Multivariable models were adjusted for the variables with *P* < .05 in univariate analysis. To estimate cumulative incidence curves and smoothed hazard rate curves, the Kaplan-Meier method and kernel-based methods were used, respectively. Two-sided *P* < .05 was considered statistically significant. Data were analyzed from December 1, 2021, through April 30, 2023.

## Results

### Patient Characteristics

We collected data from 20 405 female patients, and after exclusion, 16 251 were included in the analysis; all patients were Korean, and the mean (SD) age was 48.61 (10.06) years. The flowchart for the study population is shown in Figure 1. Among these patients, 1318 (8.11%) were classified in the younger group (age ≤35 years) and the rest (14 933 [91.89%]) in the older group (age >35 years). The clinicopathological characteristics of the whole population and the 2 age groups are summarized in the Table. The older group had a higher proportion of patients with overweight (BMI≥25) than the younger group (4108 of 14 450 [28.43%] vs 181 of 1282 [14.12%]; *P* = .04). A markedly greater proportion of patients in the younger group had a family history of breast cancer compared with the older group (151 of 1304 [11.58%] vs 1438 of 14 729 [9.76%]; *P* < .001). The younger group tended to have more aggressive features of breast cancer, such as higher histologic grade and higher proportion of T and N stage breast cancer. When we compared breast cancer subtypes, a greater proportion of the older group had HR+/*ERBB2*− subtype than the younger group (8327 [55.76%] vs 624 [47.34%]; *P* < .001) and a greater proportion had HR−/*ERBB2*− subtype in the younger group compared with the older group (341 [25.87%] vs 2481 [16.61%]; *P* < .001). A total of 1506 patients (9.27%) underwent a BRCA test, among whom 859 (57.04%) were younger patients. Although there was no statistically significant difference in *BRCA* variation status, a numerically higher percentage of patients in the younger group had *BRCA* variation than in the older group (137 of 859 [15.95%] vs 82 of 647 [12.67%]; *P* = .07).

**Table.** Baseline Characteristics of Patients at Primary Breast Cancer Surgery

Characteristic	Patients^a^			*P* value
	Total (N = 16 251)	Age at surgery, y		
		>35 (n = 14 933)	≤35 (n = 1318)	
BMI				
<18.5	580/15 732 (3.69)	423/14 450 (2.93)	157/1282 (12.25)	<.001
18.5-24.9	10 863/15 732 (69.05)	9919/14 450 (68.64)	944/1282 (73.63)	
≥25	4289/15 732 (27.26)	4108/14 450 (28.43)	181/1282 (14.12)	
Year of surgery, No. (%)				
1999-2004	3070 (18.89)	2755 (18.45)	315 (23.90)	<.001
2005-2009	5831 (35.88)	5345 (35.79)	486 (36.87)	
2010-2013	7350 (45.23)	6833 (45.76)	517 (39.23)	
Family history^b^				
No	14 444/16 033 (90.09)	13 291/14 729 (90.24)	1153/1304 (88.42)	.04
Yes	1589/16 033 (9.91)	1438/14 729 (9.76)	151/1304 (11.58)	
Histology				
IDC or DCIS	15 680/16 136 (97.17)	14 381/14 830 (96.97)	1299/1306 (99.46)	<.001
ILC or LCIS	456/16 136 (2.83)	449/14 830 (3.03)	7/1306 (0.54)	
Histologic grade				
1	919/14 114 (6.51)	869/12 980 (6.69)	50/1134 (4.41)	<.001
2	7896/14 114 (55.94)	7332/12 980 (56.49)	564/1134 (49.74)	
3	5299/14 114 (37.54)	4779/12 980 (36.82)	520/1134 (45.86)	
Nuclear grade				
1	1002/14 795 (6.77)	944/13 619 (6.93)	58/1176 (4.93)	<.001
2	8356/14 795 (56.48)	7738/13 619 (56.82)	618/1176 (52.55)	
3	5437/14 795 (36.75)	4937/13 619 (36.25)	500/1176 (42.52)	
Subtype, No. (%)				
HR+/*ERBB2*–	8951 (55.08)	8327 (55.76)	624 (47.34)	<.001
HR+/*ERBB2*+	2071 (12.74)	1888 (12.64)	183 (13.88)	
HR–/*ERBB2*+	2407 (14.81)	2237 (14.98)	170 (12.90)	
HR–/*ERBB2*–	2822 (17.37)	2481 (16.61)	341 (25.87)	
T stage, No. (%)				
Is	1459 (8.98)	1349 (9.03)	110 (8.35)	<.001
1,2	13 892 (85.48)	12 804 (85.74)	1088 (82.55)	
3,4	900 (5.54)	780 (5.22)	120 (9.10)	
N stage, No. (%)				
0	10 656 (65.57)	9860 (66.03)	796 (60.39)	<.001
≥1	5595 (34.43)	5073 (33.97)	522 (39.61)	
Radiotherapy				
No	5350/16 191 (33.04)	4909/14 875 (33.00)	441/1316 (33.51)	.71
Yes	10 841/16 191 (66.96)	9966/14 875 (67.00)	875/1316 (66.49)	
Chemotherapy				
No	6441/16 168 (39.84)	6125/14 856 (41.23)	316/1312 (24.09)	<.001
Yes	9727/16 168 (60.16)	8731/14 856 (58.77)	996/1312 (75.91)	
Hormone therapy				
No	5135/16 137 (31.82)	4598/14 827 (31.01)	537/1310 (40.99)	<.001
Yes	11 002/16 137 (68.18)	10 229/14 827 (68.99)	773/1310 (59.01)	
*BRCA* sequence variation^c^				
No	1287/1506 (85.46)	565/47 (87.33)	722/859 (84.05)	.07
Yes	219/1506 (14.54)	82/647 (12.67)	137/859 (15.95)	

Abbreviations: BMI, body mass index (calculated as weight in kilograms divided by height in meters squared); *BRCA*, breast cancer susceptibility genes 1 and 2; DCIS, ductal carcinoma in situ; HR, hormone receptor; IDC, invasive ductal carcinoma; ILC, invasive lobular carcinoma; LCIS, lobular carcinoma in situ.

^a^ Data are presented as the number/total number (percentage) of patients unless otherwise indicated.

^b^ First- and/or second-degree relative with breast cancer.

^c^ A total of 1506 patients underwent a BRCA test.

The clinicopathological characteristics of CBC in patients who developed CBC after PBC are summarized in eTable 1 in Supplement 1. The data for CBC were missing for 2 patients in the younger group and 11 in the older group; therefore, 69 patients in the younger group (5.24%) and 336 patients in the older group (2.25%) were analyzed. There were no significant differences in the histologic grade or the nuclear grade between PBC and CBC in both age groups. There was no significant difference in composition of breast cancer subtype between PBC and CBC in the younger group, and the proportion of patients with HR–/*ERBB2*– subtype was high (PBC, 25 of 69 [36.23%]; CBC, 24 of 69 [34.78%]). In the older group, there was a significant difference in breast cancer subtype, with a decrease in patients with HR+/*ERBB2*– (PBC, 99 of 336 [29.46%]; CBC, 68 of 336 [20.24%]) and an increase in patients with *ERBB2*+ (PBC, 57 of 336 [16.96%]; CBC, 84 of 336 [25.00%]) (*P* = .002). Patients of both age groups who developed CBC after PBC had a higher T and N stage of PBC compared with the T and N stage of CBC.

### Factors Associated With CBC

In both univariate analysis and multivariate analysis adjusted for family history, histology grade, subtype, T stage, and hormone therapy status of PBC, PBC surgery at younger age (≤35 years) was significantly associated with development of CBC. In univariate analysis, the hazard ratio was 2.49 (95% CI, 1.93-3.21), and in multivariate analysis, the hazard ratio was 2.10 (95% CI, 1.62-2.74). Other factors associated with increased risk of CBC were family history; higher nuclear grade; HR–/*ERBB2*– subtype; carcinoma in situ (patients with Tis-stage cancer had higher risk of developing CBC than those with invasive cancer with lesions less than 5 cm in diameter); in case of invasive cancer, higher T stage; and no treatment with hormone therapy (eTable 2 in Supplement 1).

We conducted a separate analysis of patients who underwent a BRCA test by adding *BRCA* variation status as an adjusting variable. Younger age at surgery was an independent factor associated with developing CBC regardless of *BRCA* variation status (hazard ratio, 1.74; 95% CI, 1.09-2.77) (eTable 3 in Supplement 1).

### Analysis for Cumulative Incidence of CBC

**Figure 2.**
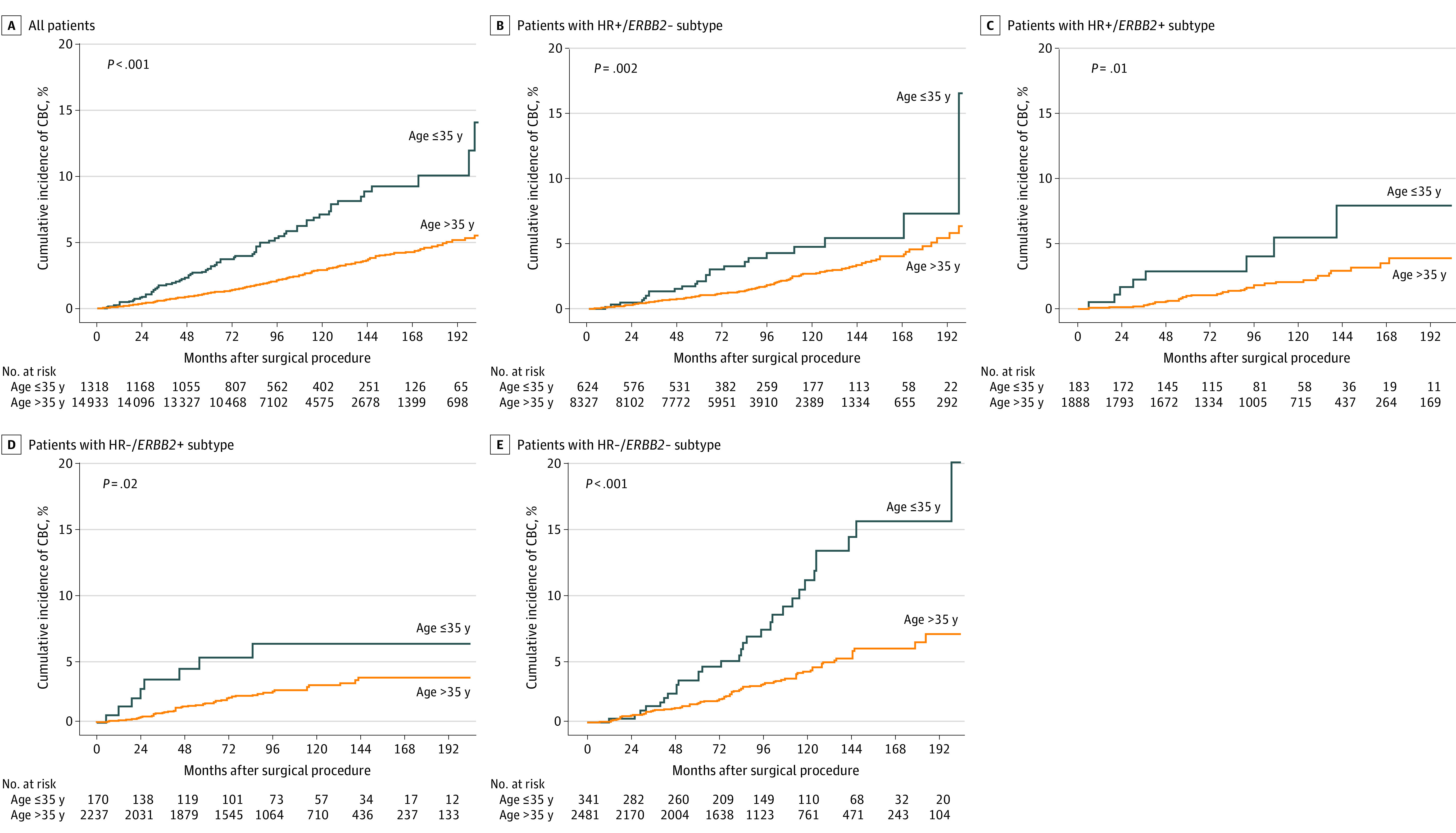
Cumulative Incidence of Developing Contralateral Breast Cancer (CBC) According to Age in All Patients and in Subgroup Analysis by Subtype HR indicates hormone receptor.

We compared the cumulative incidence of CBC in each age group. We found that 347 of 14 933 patients (2.32%) in the older group and 71 of 1318 (5.39%) in the younger group developed CBC. According to the Kaplan-Meier curve, the younger group showed significantly higher incidence of CBC than the older group (10-year cumulative CBC incidence, 7.1% vs 2.9%; *P* < .001) (Figure 2A). The median follow-up was 107 months (IQR, 79-145 months).

In subgroup analysis according to the subtype of PBC, the cumulative incidence of CBC was significantly higher in the younger group for every subtype (Figure 2B-E). The HR–/*ERBB2*– subtype was associated with the highest CBC incidence among the subtypes (10-year cumulative CBC incidence, 12.4% vs 4.4% in the younger and older group, respectively; *P* < .001). Meanwhile, the 10-year cumulative CBC incidence for the HR+/*ERBB2*– subtype was 4.8% vs 2.7% (*P* = .002) and for the HR–/*ERBB2*+ subtype was 6.1% vs 2.9% (*P* = .02) in the younger and older groups, respectively.

### Analysis for Hazard Rate of CBC

**Figure 3.**
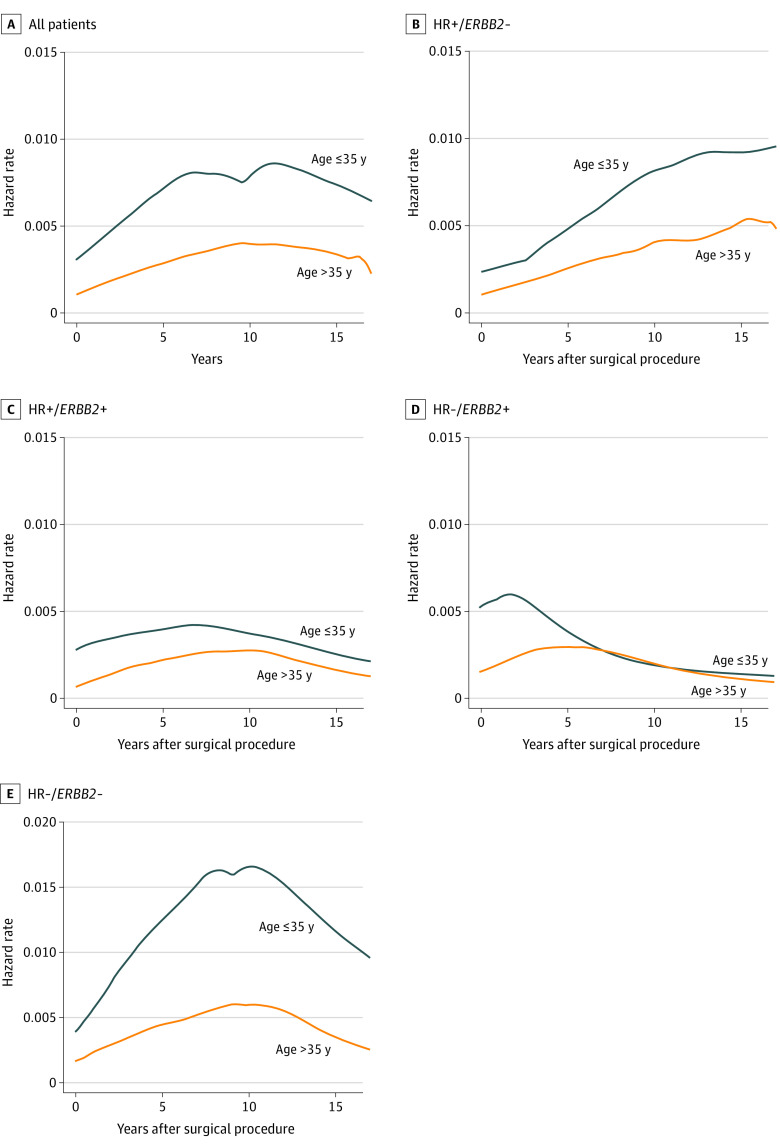
Hazard Rate for Developing Contralateral Breast Cancer (CBC) According to Age in All Patients and in Subgroup Analysis by Subtype HR indicates hormone receptor.

The younger group showed a higher hazard rate for CBC throughout the follow-up period, and both age groups presented a similar pattern of hazard rate; risk for CBC increased until the peak at around 10 years (Figure 3A). In subgroup analyses, the peak occurrence period of CBC varied according to the subtype of PBC. For the HR+/*ERBB2*– subtype, the hazard rate continuously increased over time in both age groups. For the HR–/*ERBB2*– subtype, CBC risk peaked at around 10 years. For both subtypes, younger patients showed higher hazard rates. A total of 170 of 1318 patients in the younger group (12.90%) were categorized as having the HR–/*ERBB2*+ subtype; 8 of these patients (4.71%), with median follow-up of 128 months (IQR, 87-163 months), developed CBC. For the HR–/*ERBB2*+ subtype, the hazard rate in the younger group peaked earlier, at 1.7 years, compared with 4.8 years since first surgery in the older group (Figure 3B-E).

## Discussion

In this large-scale retrospective study, the younger group, which constituted 8.11% of the total study population, had a significantly higher cumulative incidence of CBC compared with the older group, especially in those with the HR–/*ERBB2*– subtype. The CBC risk pattern showed different timing according to the subtype of PBC, and in the younger group with the HR–/*ERBB2*+ subtype, the peak occurrence time for CBC was earlier (before 3 years since surgery for PBC) than among other patients.

The cumulative incidence of CBC in the younger group was significantly higher compared with that in the older group, with a 10-year cumulative incidence of 7.1% vs 2.9%. Our findings align with those of a systematic review^13^ that reported a median 10-year cumulative incidence of 3.1% for CBC. Similarly, a nationwide Danish study^14^ reported 10-year cumulative incidences of 5.5%, 4.7%, and 4.2% for age groups younger than 45 years, 45 to 54 years, and older than 55 years, respectively, which are consistent with the CBC incidence range observed in our study.

According to large population studies^15,16^ conducted in North America and Europe, the annual incidence of CBC ranges relatively consistently from 0.2% to 0.7%, even with long follow-up, leading to a continuous increase in the cumulative incidence of CBC. We evaluated whether risk patterns at certain time frames after surgery for PBC varied according to the subtype or age at surgery. In a previous study by some of us,^9^ young patients (age <35 years) with the *ERBB2*+ subtype of PBC had the highest risk for CBC at around 4.6 years after PBC surgery, while the risk among young patients with the HR+/*ERBB2*– subtype peaked at 7.1 years; other patients showed a constant increase over the follow-up period. As that study was propensity matched for young patients (4 controls per case), we analyzed general population data in the current study. This study showed a similar result in the younger group with HR–/*ERBB2*+ subtype, with the peak hazard rate reached earlier in the younger group compared with the older group (Figure 3D). This may be interpreted as having a shorter interval for developing CBC. This was also shown in a Kaplan-Meier graph for cumulative incidence; unlike the HR+/*ERBB2*– or HR–/*ERBB2*– subtype, which showed a constant increase in cumulative incidence, the younger group with HR–/*ERBB2*+ subtype showed a relatively rapid increase at first and then a plateau afterward (Figure 2). To our knowledge, there is no study analyzing the hazard rate for CBC according to the subtype of PBC, especially according to *ERBB2* status. In our study, 170 of 1318 patients in the younger group (12.90%) were categorized as having the HR–/*ERBB2*+ subtype. In this subgroup, 8 patients (4.71%), with median follow-up of 128 months (IQR, 87-163 months), developed CBC, which was a longer observation period compared with the whole study population (median follow-up, 107 months [IQR, 79-145 months]). Incidence of CBC in this subgroup (4.7%) was less than the 10-year cumulative incidence for the whole younger group (7.1%). We can conclude that although patients with HR–/*ERBB2*+ breast cancer had a short interval for developing CBC, the incidence was lower than for other subtypes. Our findings suggest that a more thorough examination, such as breast magnetic resonance imaging for CBC, can be considered in young patients with the HR–/*ERBB2*+ subtype at an early period after the surgery for PBC, as the short interval for developing CBC is a poor prognostic factor.^17^ However, the number of patients who developed CBC in this subgroup was small; therefore, multicenter studies with larger populations are needed to further validate this result.

In our study, diagnosis of PBC at a young age was significantly associated with higher risk for CBC. Many other studies have shown similar results.^3,4^ To examine whether young patients with breast cancer are at risk for developing CBC regardless of family history or *BRCA* variation, we performed a separate multivariate Cox proportional hazards regression analysis with 1506 patients who underwent a BRCA test. Age of 35 years or younger at surgery was an independent factor associated with developing CBC regardless of *BRCA* variation status and family history, with a hazard ratio of 1.74 (95% CI, 1.09-2.77). However, patients who underwent BRCA testing were not randomized. In Korea, only patients with both ovarian and breast cancer, bilateral breast cancer, family history of breast or ovarian cancer, or a diagnosis of breast cancer before age 40 years were covered by insurance for BRCA testing until 2020. In other words, many patients with test results in this study had a higher chance of *BRCA* variation; thus, our results may not accurately represent the whole study population.

In our study, patients with Tis-stage cancer had higher risk of developing CBC than those with invasive cancer with lesions less than 5 cm in diameter (eTable 2 in Supplement 1). Similar results were found in a nationwide population study in the Netherlands,^18^ which showed that the group with ductal carcinoma in situ had slightly higher risk for CBC compared with the group with invasive carcinoma. Also, in a population study^15^ using data from the Surveillance, Epidemiology, and End Results program, diagnosis of ductal carcinoma in situ for PBC was associated with greater risk of CBC compared with invasive cancer for the first 6 years. The proposed reason for these results was more extensive use of systemic therapy, which led to a lower prevalence of CBC in patients with invasive cancer.

Our study included a relatively large population to show incidence and hazard rates for developing CBC, especially in young patients who are at higher risk. This could be valuable information for young patients seeking consultation for CBC and CPM.

### Strengths and Limitations

A strength is that the Asan Medical Center is 1 of the largest hospitals in Korea, and approximately 10% of Korean patients with breast cancer undergo surgery at the hospital. This allowed for a relatively large cross-section of the relevant population to be included in our study. Moreover, as a single-center study, there was a relatively uniform approach to pathology and treatment, which may have minimized the variability in these factors and allowed for a more focused analysis. The long follow-up period in our study further strengthens the reliability of the data.

The main limitation of this study was the relatively small number of patients with CBC, especially of those with the *ERBB2*+ subtypes in the younger group. Additionally, our study included possible selection bias owing to its retrospective and single-center nature.

## Conclusions

In this cohort study, we demonstrated that patients aged 35 years or younger with breast cancer, especially those with triple-negative breast cancer, had higher risk for developing CBC compared with older patients. Furthermore, for the HR–/*ERBB2*+ subtype, younger patients with breast cancer had a distinct timing for developing CBC compared with older patients. These findings might provide valuable information for physicians and could assist in the decision-making process for patients considering CPM. However, further research with larger sample sizes and multicenter studies are warranted to confirm and validate our results.

## Notes

Supplement 1. **eTable 1.** Baseline Characteristics of the Primary Breast Cancer and Contralateral Breast Cancer in Patients Who Developed Contralateral Breast Cancer, According to Age Group **eTable 2.** Risk for Developing Contralateral Breast Cancer **eTable 3.** Risk for Developing Contralateral Breast Cancer in BRCA-Tested Subset

Supplement 2.
Data Sharing Statement

## References

[1] Rosenberg PS, Barker KA, Anderson WF. Estrogen receptor status and the future burden of invasive and in situ breast cancers in the United States. J Natl Cancer Inst. 2015;107(9):djv159. doi:10.1093/jnci/djv159 26063794 PMC4836802

[2] Spronk I, Schellevis FG, Burgers JS, de Bock GH, Korevaar JC. Incidence of isolated local breast cancer recurrence and contralateral breast cancer: a systematic review. Breast. 2018;39:70-79. doi:10.1016/j.breast.2018.03.011 29621695

[3] Akdeniz D, Schmidt MK, Seynaeve CM, . Risk factors for metachronous contralateral breast cancer: a systematic review and meta-analysis. Breast. 2019;44:1-14. doi:10.1016/j.breast.2018.11.005 30580169

[4] Reiner AS, John EM, Brooks JD, . Risk of asynchronous contralateral breast cancer in noncarriers of BRCA1 and BRCA2 mutations with a family history of breast cancer: a report from the Women’s Environmental Cancer and Radiation Epidemiology Study. J Clin Oncol. 2013;31(4):433-439. doi:10.1200/JCO.2012.43.2013 23269995 PMC3731919

[5] Kurian AW, McClure LA, John EM, Horn-Ross PL, Ford JM, Clarke CA. Second primary breast cancer occurrence according to hormone receptor status. J Natl Cancer Inst. 2009;101(15):1058-1065. doi:10.1093/jnci/djp181 19590058 PMC2720990

[6] Bernstein JL, Lapinski RH, Thakore SS, Doucette JT, Thompson WD. The descriptive epidemiology of second primary breast cancer. Epidemiology. 2003;14(5):552-558. doi:10.1097/01.ede.0000072105.39021.6d 14501270

[7] Vichapat V, Gillett C, Fentiman IS, Tutt A, Holmberg L, Lüchtenborg M. Risk factors for metachronous contralateral breast cancer suggest two aetiological pathways. Eur J Cancer. 2011;47(13):1919-1927. doi:10.1016/j.ejca.2011.05.004 21658939

[8] Chen Y, Semenciw R, Kliewer E, Shi Y, Mao Y. Incidence of second primary breast cancer among women with a first primary in Manitoba, Canada. Breast Cancer Res Treat. 2001;67(1):35-40. doi:10.1023/A:1010665603732 11518464

[9] Yoon TI, Kwak BS, Yi OV, . Age-related risk factors associated with primary contralateral breast cancer among younger women versus older women. Breast Cancer Res Treat. 2019;173(3):657-665. doi:10.1007/s10549-018-5031-4 30377870

[10] Graeser MK, Engel C, Rhiem K, . Contralateral breast cancer risk in BRCA1 and BRCA2 mutation carriers. J Clin Oncol. 2009;27(35):5887-5892. doi:10.1200/JCO.2008.19.9430 19858402

[11] Davies KR, Cantor SB, Brewster AM. Better contralateral breast cancer risk estimation and alternative options to contralateral prophylactic mastectomy. Int J Womens Health. 2015;7:181-187. doi:10.2147/IJWH.S52380 25678823 PMC4324540

[12] Kim J, Hong S, Lee JJ, . Analysis of the tumor characteristics in young age breast cancer patients using collaborative stage data of the Korea Central Cancer Registry. Breast Cancer Res Treat. 2021;187(3):785-792. doi:10.1007/s10549-021-06107-9 33604714

[13] Kang SY, Kim YS, Kim Z, ; Korean Breast Cancer Society. Breast cancer statistics in Korea in 2017: data from a breast cancer registry. J Breast Cancer. 2020;23(2):115-128. doi:10.4048/jbc.2020.23.e24 32395372 PMC7192743

[14] Rasmussen CB, Kjær SK, Ejlertsen B, . Incidence of metachronous contralateral breast cancer in Denmark 1978-2009. Int J Epidemiol. 2014;43(6):1855-1864. doi:10.1093/ije/dyu202 25326461

[15] Giannakeas V, Lim DW, Narod SA. The risk of contralateral breast cancer: a SEER-based analysis. Br J Cancer. 2021;125(4):601-610. doi:10.1038/s41416-021-01417-7 34040177 PMC8368197

[16] Langballe R, Frederiksen K, Jensen MB, . Mortality after contralateral breast cancer in Denmark. Breast Cancer Res Treat. 2018;171(2):489-499. doi:10.1007/s10549-018-4846-3 29948403

[17] Liederbach E, Wang CH, Lutfi W, . Survival outcomes and pathologic features among breast cancer patients who have developed a contralateral breast cancer. Ann Surg Oncol. 2015;22(suppl 3):S412-S421. doi:10.1245/s10434-015-4835-2 26334294

[18] Giardiello D, Kramer I, Hooning MJ, . Contralateral breast cancer risk in patients with ductal carcinoma in situ and invasive breast cancer. NPJ Breast Cancer. 2020;6(1):60. doi:10.1038/s41523-020-00202-8 33298933 PMC7609533

