# Supplementary material for: Age-Related Incidence and Peak Occurrence of Contralateral Breast Cancer
Source: JAMA Netw Open. 2023 Dec 15;6(12):e2347511. doi: 10.1001/jamanetworkopen.2023.47511 (PMC10724757; doi:10.1001/jamanetworkopen.2023.47511)
Supplement: Supplement 1. — eTable 1. Baseline Characteristics of the Primary Breast Cancer and Contralateral Breast Cancer in Patients Who Developed Contralateral Breast Cancer, According to Age Group eTable 2. Risk for Developing Contralateral Breast Cancer eTable 3. Risk for Developing Contralateral Breast Cancer in BRCA-Tested Subset [file jamanetwopen-e2347511-s001.pdf]

Supplementary Online Content

Kim H, Yoon TI, Kim S, et al. Age-related incidence and peak occurrence of contralateral breast cancer. *JAMA Netw Open*. 2023;6(12):e2347511.
10.1001/jamanetworkopen.2023.47511

eTable 1. Baseline Characteristics of the Primary Breast Cancer and Contralateral Breast Cancer in Patients Who Developed Contralateral Breast Cancer, According to Age Group

eTable 2. Risk for Developing Contralateral Breast Cancer

eTable 3. Risk for Developing Contralateral Breast Cancer in BRCA-Tested Subset

This supplementary material has been provided by the authors to give readers additional information about their work.

© 2023 Kim H et al. *JAMA Network Open*.

eTable 1. Baseline characteristics of the primary breast cancer and contralateral breast cancer in patients who developed contralateral breast cancer, according to age group.

	Age at surgery ≤ 35			Age at surgery > 35		
	CBC patients (N=69), No. (%)			CBC patients (N=336) No. (%)		
	**PBC**	**CBC**	**P-value**	**PBC**	**CBC**	**P-value**
**Median Age ± SD**	31.57±2.64	37.33±4.19	<.001	47.21±8.33	53.07±8.92	<.001
**Histologic grade**						
1 or 2	23 (42.59)	27 (50)	0.35	129 (57.85)	128 (57.4)	0.91
3	31 (57.41)	27 (50)		94 (42.15)	95 (42.6)	
**Nuclear grade**						
1 or 2	35 (53.03)	35 (53.03)	1.00	191 (63.04)	192 (63.37)	0.92
3	31 (46.97)	31 (46.97)		112 (36.96)	111 (36.63)	
**Subtype**						
HR+/HER2-	11 (23.4)	14 (29.79)	0.72	99 (45.62)	68 (31.34)	0.002
HR+/HER2+	4 (8.51)	3 (6.38)		22 (10.14)	39 (17.97)	
HR-/HER2+	7 (14.89)	6 (12.77)		35 (16.13)	45 (20.74)	
HR-/HER2-	25 (53.19)	24 (51.06)		61 (28.11)	65 (29.95)	
**Stage_T**						
0 (Tis)	5 (7.25)	13 (18.84)	0.016	53 (15.96)	92 (27.71)	<.001
1, 2	59 (85.51)	54 (78.26)		258 (77.71)	232 (69.88)	
3, 4	5 (7.25)	2 (2.9)		21 (6.33)	8 (2.41)	
**Stage_N**						
0	38 (59.38)	57 (89.06)	<.001	238 (73.23)	279 (85.85)	<.001
≥1	26 (40.63)	7 (10.94)		87 (26.77)	46 (14.15)	
**Radiotherapy**						
No	28 (41.18)	19 (27.94)	0.05	127 (39.44)	99 (30.75)	0.005
Yes	40 (58.82)	49 (72.06)		195 (60.56)	223 (69.25)	
**Chemotherapy**						
No	8 (11.76)	34 (50)	<.001	151 (47.04)	211 (65.73)	<.001
Yes	60 (88.24)	34 (50)		170 (52.96)	110 (34.27)	
**Hormone therapy**						
No	35 (52.24)	36 (53.73)	0.84	142 (44.38)	131 (40.94)	0.30
Yes	32 (47.76)	31 (46.27)		178 (55.63)	189 (59.06)	

© 2023 Kim H et al. *JAMA Network Open*.

PBC, primary breast cancer; CBC, contralateral breast cancer; SD, standard deviation; HR, hormone receptor; HER2, human epidermal growth factor receptor 2.

© 2023 Kim H et al. *JAMA Network Open*.

eTable 2. Risk for developing contralateral breast cancer.

		Univariate				Multivariate[a]				Multivariate[b]			
		Hazard ratio	95% CI		p-value	Hazard ratio	95% CI		p-value	Hazard ratio	95% CI		p-value
**Age at surgery**	≤35	2.487	1.927	3.211	<0.0001	2.159	1.659	2.810	<0.0001	2.104	1.615	2.741	<0.0001
**BMI (kg/m^2)**	<18.5	1.598	1.045	2.444	0.030	1.307	0.848	2.015	0.225	1.288	0.835	1.986	0.252
	18.5–24.9	1			0.056	1			0.377	1			0.427
	≥25	0.919	0.733	1.151	0.461	0.947	0.754	1.189	0.638	0.953	0.759	1.197	0.681
**Calendar Period (Year of surgery)**	1999–2004	1			0.539								
	2005–2009	0.991	0.779	1.260	0.940								
	2010–2013	0.870	0.654	1.155	0.335								
**Family history[c]**	Yes	1.955	1.507	2.537	<0.0001	1.902	1.465	2.468	<0.0001	1.896	1.461	2.461	<0.0001
**Histology grade**	1 or 2	1											
	3	1.530	1.259	1.860	0.000	1.409	1.112	1.784	0.005	1.374	1.083	1.743	0.009
**Nuclear grade**	1 or 2	1											
	3	1.333	1.093	1.625	0.004								
**Subtype**	HR+/HER2-	1			<0.001	1			<0.0001	1			0.010
	HR+/HER2+	0.896	0.648	1.239	0.505	0.834	0.601	1.157	0.278	0.823	0.593	1.142	0.245
	HR-/HER2+	1.084	0.809	1.453	0.588	0.971	0.713	1.321	0.850	0.673	0.440	1.027	0.067
	HR-/HER2-	1.941	1.543	2.441	0.000	1.632	1.251	2.129	0.000	1.119	0.750	1.670	0.582
**T Stage**	Tis	1.691	1.279	2.235	0.000	2.147	1.599	2.884	<0.0001	1.840	1.331	2.544	0.000

© 2023 Kim H et al. *JAMA Network Open*.

	1, 2	1			<0.001	1			<0.0001	1			<0.0001
	3, 4	1.770	1.196	2.621	0.004	1.743	1.175	2.587	0.006	1.762	1.188	2.614	0.005
**N Stage**	0	1											
	≥1	0.874	0.708	1.079	0.211								
**Hormone therapy**	Yes	0.538	0.443	0.653	0.000					0.635	0.446	0.905	0.012
**Radiotherapy**	Yes	0.865	0.710	1.054	0.151								
**Chemotherapy**	Yes	0.981	0.806	1.195	0.849								

[a]adjusted variables: age at surgery, BMI, family history, histology grade, subtype, T stage

[b]adjusted variables: age at surgery, BMI, family history, histology grade, subtype, T stage, hormone therapy status

[c]Family history: First- and second-degree relative breast cancer family history

BMI: Body mass index, HR: Hormone receptor, HER2: Human epidermal growth factor receptor 2, CI: Confidence inteerval

© 2023 Kim H et al. *JAMA Network Open*.

eTable 3. Risk for developing contralateral breast cancer in BRCA-tested subset. (N= 1,506)

		Univariate				Multivariate[a]				Multivariate[b]			
		Hazard ratio	95% CI		p-value	Hazard ratio	95% CI		p-value	Hazard ratio	95% CI		p-value
**Age at surgery**	≤35	2.082	1.378	3.144	0.000	1.739	1.091	2.772	0.020	1.739	1.091	2.772	0.020
**BMI (kg/m^2)**	<18.5	1.680	0.889	3.173	0.110	1.360	0.710	2.606	0.354	1.360	0.710	2.606	0.354
	18.5–24.9	1			0.088	1			0.261	1			0.261
	≥25	0.670	0.363	1.236	0.200	0.682	0.367	1.267	0.226	0.682	0.366	1.268	0.226
**Calendar Period (Year of surery)**	1999–2004	1			0.539								
	2005–2009	0.761	0.471	1.230	0.265								
	2010–2013	0.868	0.466	1.619	0.657								
**Family history[c]**	Yes	0.554	0.352	0.872	0.011	0.640	0.385	1.064	0.086	0.640	0.385	1.064	0.086
**Histology grade**	1 o r2	1											
	3	1.840	1.228	2.757	0.003	1.314	0.781	2.211	0.304	1.314	0.781	2.212	0.304
**Nuclear grade**	1 or 2	1											
	3	1.542	1.026	2.319	0.037								
**Subtype**	HR+/HER2-	1			0.003	1			0.668	1			0.713
	HR+/HER2+	1.497	0.775	2.891	0.230	1.450	0.745	2.825	0.275	1.451	0.745	2.827	0.274
	HR-/HER2+	1.406	0.712	2.776	0.327	1.267	0.612	2.623	0.524	1.276	0.517	3.149	0.597
	HR-/HER2-	2.398	1.505	3.821	0.000	1.323	0.731	2.395	0.355	1.333	0.589	3.017	0.490
**T Stage**	Tis	1.664	0.884	3.135	0.115	2.318	1.162	4.625	0.017	2.327	1.109	4.882	0.026

© 2023 Kim H et al. *JAMA Network Open*.

	1, 2	1			0.080	1			0.029	1			0.041
	3, 4	2.003	0.923	4.348	0.079	1.698	0.772	3.735	0.188	1.699	0.772	3.736	0.188
**N Stage**	0	1											
	≥1	1.317	0.872	1.988	0.191								
**Hormone therapy**	Yes	0.541	0.360	0.813	0.003					1.009	0.516	1.974	0.979
**Radiotherapy**	Yes	0.865	0.710	1.054	0.151								
**Chemotherapy**	Yes	0.981	0.806	1.195	0.849								
**BRCA mutation**	Yes	4.446	2.926	6.758	<0.0001	4.715	2.955	7.524	<0.0001	4.714	2.953	7.524	<.00001

[a]adjusted variables: age at surgery, BMI, family history, histology grade, subtype, T stage, BRCA mutation status

[b]adjusted variables: age at surgery, BMI, family history, histology grade, subtype, T stage, BRCA mutation status, hormone therapy status,

[c]Family history: First- and second-degree relative breast cancer family history

BMI: Body mass index, HR: Hormone receptor, HER2 : Human epidermal growth factor receptor 2, BRCA: breast cancer gene 1, 2

© 2023 Kim H et al. *JAMA Network Open*.
