# Supplementary material for: Age-Related Incidence and Peak Occurrence of Contralateral Breast Cancer
Source: JAMA Netw Open. 2023 Dec 15;6(12):e2347511. doi: 10.1001/jamanetworkopen.2023.47511 (PMC10724757; doi:10.1001/jamanetworkopen.2023.47511)
Supplement: Supplement 2. — Data Sharing Statement [file jamanetwopen-e2347511-s002.pdf]

Data Sharing Statement

Kim. Age-Related Incidence and Peak Occurrence of Contralateral Breast Cancer. *JAMA Netw Open*. Published December 15, 2023. doi:10.1001/jamanetworkopen.2023.47511

Data
Data available: No
